# Rare Association between Two Genetic Conditions: Turner Syndrome and Neurofibromatosis Type 1

**DOI:** 10.1155/2022/6116603

**Published:** 2022-04-15

**Authors:** R. El Qadiry, K. Danaoui, H. Nassih, A. Bourrahouat, I. Ait Sab

**Affiliations:** Pediatric B Department, Mother-Child Pole, Mohammed VI University Hospital, Marrakesh, Morocco

## Abstract

Turner's syndrome (TS) is a sex chromosome disorder due to loss of either all or part of the X chromosome, in some or all the cells of the body. Neurofibromatosis type 1 (NF-1) is a multisystemic genetic disorder that is only rarely observed in association with Turner syndrome. Only six cases of Turner syndrome associated with NF-1 have been reported in the literature. In this study, we report the first case with TS and NF-1 in a Moroccan child. *Case Report*. A 16-year-old female was born of a nonconsanguineous marriage. In her family history, her mother had multiple café-au-lait spots with Lisch nodules on ophthalmologic examination. She was diagnosed with TS (karyotype: 45, X) due to short stature and characteristic features. The diagnosis of NF-1 was made according to the presence of four diagnostic criteria of the National Institute of Health Consensus Development Conference. *Conclusion*. Coexistence of NF-1 with TS is rare. We consider that this may be the seventh case report of TS associated with NF-1.

## 1. Introduction

Turner's syndrome (TS) occurs due to loss of either all or part of the X chromosome, in some or all the cells of the body with an incidence of at least 1 in 2500 live births. Short stature is a cardinal finding in all girls with TS [1].

Neurofibromatosis type 1 (NF-1) is an autosomal dominant disorder due to loss-of-function mutations in the tumor suppressor NF-1 gene. It is characterized particularly by café-au-lait spots and fibromatous tumors of the skin [2].

NF-1 is rarely observed in association with Turner syndrome. Only six cases of TS associated with NF-1 have been reported in the literature. In this study, we report the first case with TS and NF-1 in a Moroccan child.

## 2. Case

**Figure 1:**
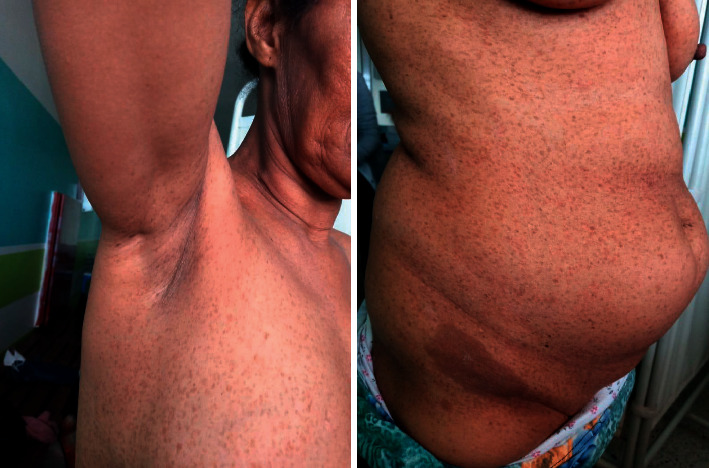
Hyperpigmented skin macules with multiple café-au-lait spots, axillary, and inguinal freckling in her mother.

A 16-year-old female was admitted to our department for evaluation of short stature and delayed puberty. She was born of a nonconsanguineous marriage, after an uncomplicated pregnancy via vaginal delivery. Psychomotor development was delayed. There was a positive history of congenital lymphedema and recurrent acute otitis media with normal hearing assessment. In her family history, her mother had multiple café-au-lait spots (Figure 1) with Lisch nodules on ophthalmologic examination.

**Figure 2:**
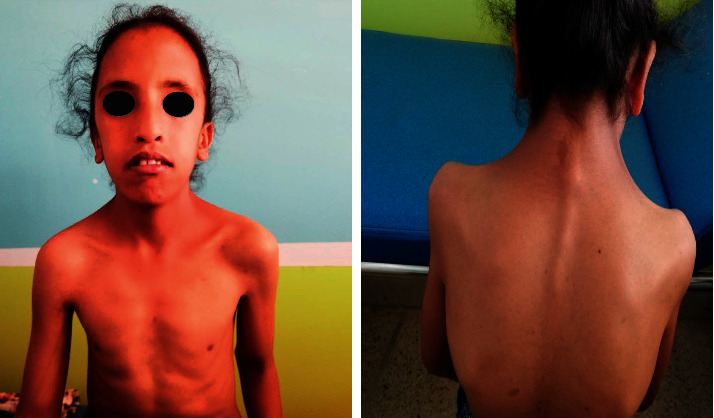
Characteristic appearance with webbed neck, shielded chest, and a low posterior hair line.

**Figure 3:**
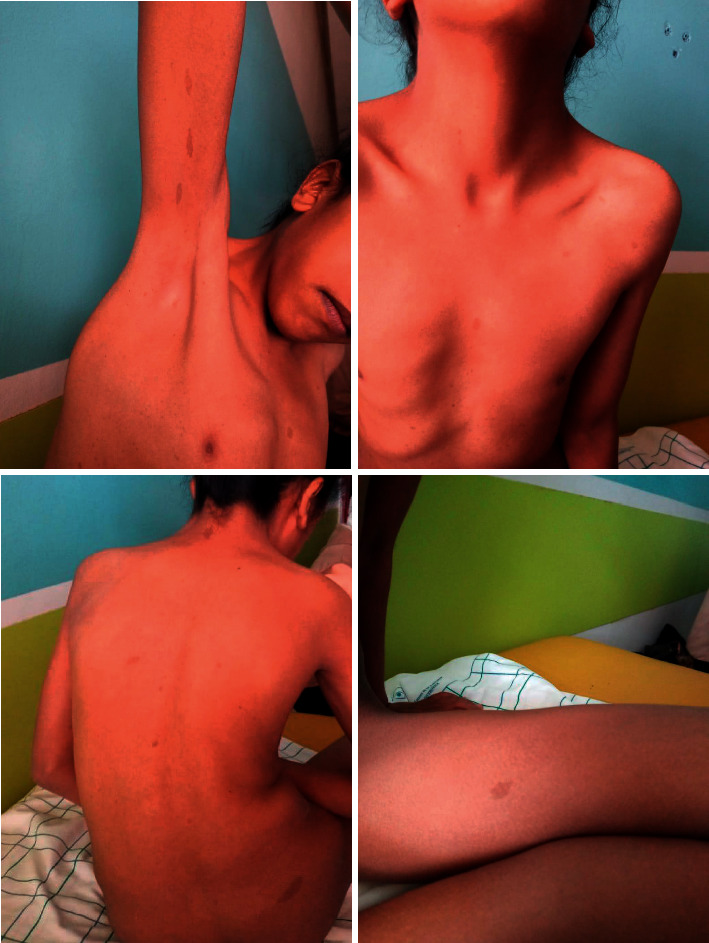
Hyperpigmented skin macules with multiple café-au-lait spots and axillary freckling in our patient.

The patient's weight was 21 kg (greater than 4 SD) and her height was 120 cm (greater than 4 SD). The physical examination revealed a webbed neck, shielded chest, a low posterior hair line, and scoliosis (Figure 2). She had stage 1 for both breast and pubic hair development on Tanner staging. Dermatological examination revealed more than 10 café-au-lait spots with diameter >0.5 cm, axillary and inguinal freckling (Figure 3). The patient did not have any neurofibroma. The mucous membranes were not affected. The rest of the clinical examinations were normal.

Investigations were ordered to confirm Turner syndrome as well as neurofibromatosis type 1. The primary laboratory profile included a complete blood count, fasting blood sugar, blood urea nitrogen and creatinine, liver function test, and lipid profile and was normal. Her FSH was 154.90 IU/l and LH was 27.4 IU/l. A karyotype analysis was done because the Turner stigmata revealed 45, X0.

**Figure 4:**
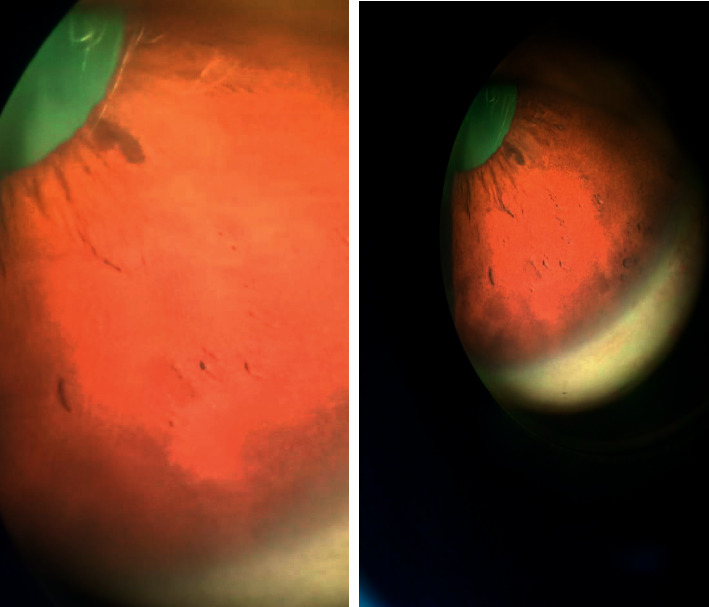
Lisch nodules without clinical visual involvement on ophthalmologic examination.

The pertinent paraclinical studies, including echocardiography, celiac serology, and thyroid function test, were normal. Ultrasonography of the abdomen showed agenesis of gonads with hypoplastic uterus. Kidneys, ureters, and bladder were normal. Skeletal maturation, evaluated by a left wrist X-ray according to the technique of Greulich and Pyle, was 15 years. Serum IGF-1 level was within normal limits. A brain and pituitary MRI to exclude pituitary lesions or structural abnormalities showed normal results. Her eyes showed Lisch nodules without clinical visual involvement during ophthalmologic examinations (Figure 4).

A final diagnosis of Turner syndrome with neurofibromatosis type 1 was made according to the presence of four diagnostic criteria of the National Institute of Health Consensus Development Conference.

Because the patient had an advanced bone age (15 years) and the high risk of malignancy development, GH therapy was not administered. Hence, she was treated with estrogen replacement therapy, with periodic evaluations.

## 3. Discussion

There is a well-known association between NF-1 and Noonan syndrome-like manifestations, but there are a few cases reporting an association between NF-1 and Turner syndrome. Our patient is the seventh reported with clinical features of both NF-1 and TS.

Neurofibromatosis type 1 (NF-1) is the most common among the forms of neurofibromatosis (NF). Clinically, NF-1 is characterized by the presence of multiple (>6) café-au-lait spots and at least one of the following diagnostic criteria of the National Institute of Health Consensus Development Conference: axillary or groin freckling, Lisch nodules, optic glioma, cutaneous or subcutaneous neurofibromas, or first-degree relative with an NF-1 diagnosis. Our patient presents four signs of NF-1 [3].

NF-1 represents a major risk factor for development of malignancy, particularly malignant peripheral nerve sheath tumors and optic gliomas, and malignancy is an important component of the NF-1 phenotype and one of the few potentially fatal complications [4].

NF-1 is often associated with endocrine abnormalities and short stature. These abnormalities have been generally attributed to the presence of suprasellar or other brain lesions, which may cause growth hormone (GH) deficiency (GHD), scoliosis, and other skeletal problems that may interfere with normal skeletal development. In our patient, these risk factors were explored, and there was no GHD or intracranial lesion, except for mild scoliosis [5].

Turner syndrome is defined by a combination of phenotypic features that vary throughout a patient's life. The most common presentation for TS is short stature. The guidelines recommend screening if the girl has an unexplained short stature. Due to characteristic features like webbing of neck, low hair line, widely spaced nipple, and lack of breast development with elevated follicle-stimulating hormone levels with short stature, Turner syndrome was suspected and confirmed in our patient [6].

Earlier diagnosis of Turner syndrome has been shown to give the best chance for effective treatment and thus good clinical outcomes. It allows the timely introduction of recombinant human growth hormone (rhGH) to improve the height prior to estrogen therapy. Unfortunately, the diagnosis in our patient is made at the age of sixteen years; the time gap to initiate rhGH therapy is missed [6].

Finally, there has been a long-standing concern about the safety of GH treatment in NF-1 patients. It has been pointed out that rhGH treatment, at the dose recommended in GH deficiency, is well tolerated and does not influence the incidence of intracranial tumor development in NF-1 patients. Although these observations on a large number of patients are reassuring, there remains the problem of the high dose of rhGH recommended in Turner syndrome with inherent risk of malignancy [7].

## 4. Conclusion

Coexistence of NF-1 with TS is rare. Thus, these disorders may be overlapping. We therefore emphasize that each patient with Turner-like symptoms should be carefully examined for café-au-lait macules. The decision to treat with GH a condition such as NF-1, generally associated with inherent risk of malignancy, is challenging, especially in our context.

## Data Availability

No data were used to support this study.
